# Adenovirus Type 6: Subtle Structural Distinctions from Adenovirus Type 5 Result in Essential Differences in Properties and Perspectives for Gene Therapy

**DOI:** 10.3390/pharmaceutics13101641

**Published:** 2021-10-08

**Authors:** Margarita Romanenko, Ivan Osipov, Sergey V. Netesov, Julia Davydova

**Affiliations:** 1 Department of Surgery, University of Minnesota, Minneapolis, MN 55455, USA; 2 Laboratory of Biotechnology and Virology, Department of Natural Sciences, Novosibirsk State University, 630090 Novosibirsk, Russia; i.osipov6@g.nsu.ru (I.O.); netesov.s@nsu.ru (S.V.N.); 3 Masonic Cancer Center, University of Minnesota, Minneapolis, MN 55455, USA; 4 Center for Genome Engineering, University of Minnesota, Minneapolis, MN 55455, USA

**Keywords:** adenovirus, serotype 6, Ad6, Ad5, oncolytic viruses, virotherapy, vaccine, immunotherapy, Kupffer cells, liver tropism

## Abstract

Adenovirus vectors are the most frequently used agents for gene therapy, including oncolytic therapy and vaccine development. It’s hard to overestimate the value of adenoviruses during the COVID-19 pandemic as to date four out of four approved viral vector-based SARS-CoV-2 vaccines are developed on adenovirus platform. The vast majority of adenoviral vectors are based on the most studied human adenovirus type 5 (HAdV-C5), however, its immunogenicity often hampers the clinical translation of HAdV-C5 vectors. The search of less seroprevalent adenovirus types led to another species C adenovirus, Adenovirus type 6 (HAdV-C6). HAdV-C6 possesses high oncolytic efficacy against multiple cancer types and remarkable ability to induce the immune response towards carrying antigens. Being genetically very close to HAdV-C5, HAdV-C6 differs from HAdV-C5 in structure of the most abundant capsid protein, hexon. This leads to the ability of HAdV-C6 to evade the uptake by Kupffer cells as well as to distinct opsonization by immunoglobulins and other blood proteins, influencing the overall biodistribution of HAdV-C6 after systemic administration. This review describes the structural features of HAdV-C6, its interaction with liver cells and blood factors, summarizes the previous experiences using HAdV-C6, and provides the rationale behind the use of HAdV-C6 for vaccine and anticancer drugs developments.

## 1. Introduction

Adenovirus (Ad) is one of the most extensively used viral vectors in the field of gene and oncolytic viral therapies. According to the Wiley database, a total of 573 Ad-based therapy trials have taken place since 1993, which accounts for 27% of all applicable viral vectors [1]. It is hard to overestimate the value of Ad during the current pandemic, as all four SARS-CoV-2 viral vector vaccines approved for full or limited use are based on adenoviral vectors, and more are coming down the pipeline [2,3,4,5].

Multiple properties of Ad make this agent an attractive vector for the development of vaccines and therapies for genetic diseases and cancer. First, Ad has a stable double-stranded DNA genome, which does not integrate into host DNA, conferring minimal potential risk of insertional mutagenesis [6]. Second, Ad can target a broad spectrum of both dividing and non-dividing cells [7,8]. Third, Ads can be efficiently produced at high titer, and its manufacturing is well-established [6,8]. Fourth, Ads have a comparatively large packaging capacity, allowing insertion of a variety of therapeutic and imaging transgenes [9,10]. Furthermore, the Ad genome and its replication machinery have been highly characterized, which allows fine-tuning of purpose-built Ad vectors. This includes retargeting of Ad to specific cell types by changing particular Ad genes or regulatory sequences [7].

Human adenoviruses (HAdVs) belong to the *Mastadenovirus* genus of the *Adenoviridae* family and are comprised of 104 types which are classified into seven species (from A to G) [11]. Human adenovirus type 5 (HAdV-C5) is the most widely utilized virus in this family with its applications ranging from gene delivery to vaccination and cancer therapy. Since its isolation from adenoid tissues in 1953, HAdV-C5 became the most popular tool for therapeutic development with commercially available kits and technologies allowing easy cloning and vector production.

HAdV-C5-based vectors proved themselves as powerful oncolytic cancer agents as they can effectively replicate and lyse tumor cells [12,13]. Furthermore, the recent discovery of oncolytic viruses facilitating immunogenic cell death, which evokes a local antitumor response, has opened a new avenue for its use in the clinic [14]. Yet, the HAdV-C5-based vectors have been often criticized for a number of drawbacks, mainly for reported liver tropism and immunogenicity, which undoubtedly limit the clinical and commercial value of HAdV-C5 [15]. It is also important to acknowledge HAdV-C5 high seroprevalence, which may result in rapid clearance of adenoviral virions from blood stream by neutralizing antibodies and inability to treat a systemic disease. Indeed, a considerable number of human patients have faced an HAdV-C5 infection in their lifetime and developed anti-HAdV-C5 neutralizing antibodies (nAb) [16]. These same antibodies can hamper the desired effects of HAdV-C5-based vector therapies or vaccinations. All these obstacles have led to the search of less seroprevalent adenovirus types for vector development.

Among all the alternative Ads studied for their potential therapeutic value, human adenovirus type 6 (HAdV-C6) is a highly promising candidate. HAdV-C5 and HAdV-C6 (for text simplification will be referred as Ad5 and Ad6, respectively) belong to the same species (C), sharing a high degree of sequence homology [17]. However, subtle differences in the Ad6 genome greatly affect its features and possible use. Here, we discuss the biological properties of this specific type and prospects for Ad6 application for cancer therapy and vaccine development.

## 2. Comparative Structure of Ad6 versus Ad5

**Figure 1:**
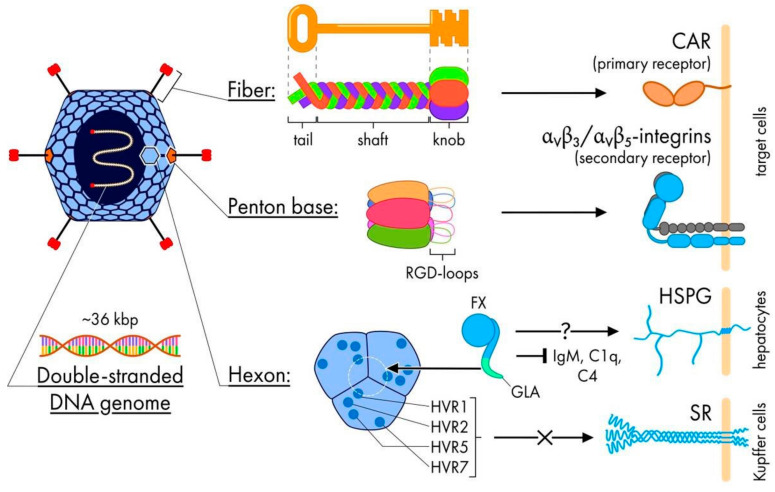
Ad6 structure and function of three main capsid proteins. The Ad6 fiber is the first main capsid protein which is responsible for interaction with target cells. The interaction happens via binding of the Ad6 fiber knob to Coxsackie and Adenovirus receptor (CAR). Next, the Penton base directly binds to αvβ5/αvβ3 integrins, leading to the internalization of Ad6. The γ-carboxyglutamic acid (GLA) domain of the Hexon protein binds to blood factors (e.g., coagulation factor X (FX)). FX protects virions from natural IgM and complement and can bridge coated viral particles to hepatocyte Heparan Sulfate Proteoglycans (HSPGs) promoting hepatocyte transduction, which is especially important upon systemic administration. The overall low charge of hexon hypervariable regions (HVRs) results in poor Ad6 uptake by scavenger receptors (SR) on Kupffer cell and other macrophages.

Similar to all members of the Ad family, both Ad5 and Ad6 are non-enveloped, double-stranded DNA viruses with an icosahedral capsid comprised of three major proteins–fiber, penton and hexon (Figure 1).

**Figure 2:**
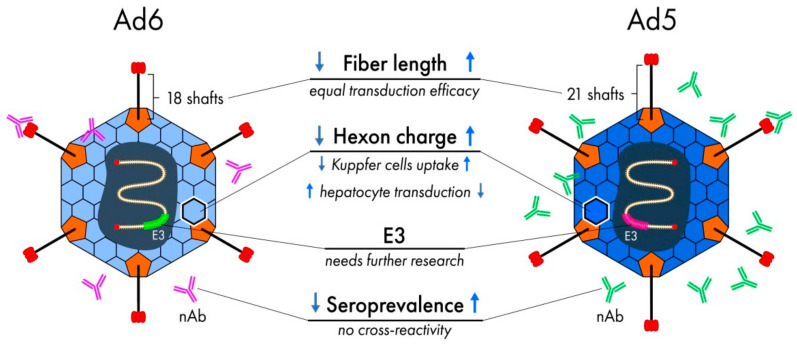
Major differences between Ad6 and Ad5. (1) The fiber protein of Ad6 has 18 shaft repeats in contrast to 21 repeats for Ad5. (2) Hexon charge of Ad6 is lesser than that of Ad5. (3) The Ad6 E3 region significantly differs from Ad5 E3. (4) Ad6 is less seroprevalent than Ad5, and there is no neutralizing antibodies (nAb) cross-reactivity between these two types.

The first viral protein to interact with the host cell is the Ad fiber. This trimeric protein protrudes from the Ad capsid and serves as the “first key” for Ad to “unlock” the cell membrane and enter the cytoplasm. The Ad fiber has three distinct regions: (1) the C-terminal knob domain, which is presented on the distal end of the fiber and responsible for binding to the primary cell receptor; (2) the central fiber shaft consisting of several β-turn repeats; and (3) a tail anchored in the penton base (Figure 1) [18]. In the same way as the unique pattern of a key bit determines what kind of lock can be unlocked, the fiber knob structure dictates what specific surface cell protein can be used for entering the cell. The Ad6 fiber knob utilizes the same Coxsackie and Adenovirus Receptor (CAR) as Ad5, and in general, both Ad6 knob and tail share quite high amino acid homology with Ad5 (68.89% and 100%, respectively) [19]. The fiber region that mostly distinguishes Ad6 fiber from fibers of other species C Ads (Ad5, Ad2 and Ad1) is the fiber shaft (Figure 2). The Ad6 shaft has only 18 repeats versus 21 repeats for other species C Ads, which results from the deletion of repeats 15, 16 and 17 [17]. The Ad6 fiber also has slight differences in shaft motifs 3 and 21 compared to Ad5 [19], which may alter its interaction with its receptor as these repeats are critical for fiber flexibility [20].

The second Ad protein essential for entering the cell is the pentameric penton base. The penton base plays a role as the secondary key for all human Ads except Species F Ads. The Arginine-Glycine-Aspartate (RGD) loops of the penton base are responsible for the interaction with cellular αvβ-class integrins, the secondary receptors (Figure 1). This interaction initiates endocytosis and subsequent viral particle entry [21,22]. Only minor differences have been found between Ad5 and Ad6 penton base amino acid sequences [17], suggesting they have similar affinity toward integrins.

The third major Ad capsid protein, hexon, constitutes the most abundant building material for the virus capsid. Hexon has been shown to play an important role in trafficking Ads after systemic injection in vivo and has two notable characteristics related to this function. First, it is responsible for transduction of various cell types, especially tissue macrophages, and decreases, if not eliminates, the role of Ad primary and secondary receptors after systemic delivery (Figure 1) [23,24,25]. Second, as hexon is the most profuse surface protein, it is the main target for neutralizing antibodies (nAb), which is a big obstacle for intravenously injected Ad vectors. Each subunit of trimeric hexon has seven hypervariable regions (HVRs), which differ widely in sequence even among closely related types. Amino acid sequence alignment of Ad5 and Ad6 shows high divergence in the hexon region, especially in HVRs, which results in different seroprevalence and levels of liver transduction as we will discuss below (Figure 2).

## 3. CAR as the Primary Receptor for Ad6 and Its Role for Ad Clinical Implementation

There are both advantages as well as limitations for Ads utilizing CAR as a primary receptor. CAR is known as a component of tight junctions in epithelial cells, but its function is not restricted to maintenance of the cellular connection. It also coordinates cell–cell adhesion under homeostatic conditions and is involved in pathological states including cancer development [26]. Ad6 exploits CAR to enter and subsequently lyse respiratory epithelial cells, causing the common cold [17,27]. It is interesting to note that because tight junctions localize in basolateral, but not apical, surfaces of polarized epithelial cells, Ad6 and other viruses are hindered in their access to bind their primary receptors and infect these cells. However, there is a particular isoform of CAR which does localize on apical surfaces and can be used as a receptor for Ad entering from the airway lumen [28]. Additionally, the apical localization of this CAR isoform is facilitated by pro-inflammatory cytokines produced by alveolar macrophages after endocytosis of Ads [29]. This natural ability of Ad6 to infect airway epithelium has been successfully exploited in developing intranasal vaccines, including those against influenza [30], Ebola [31] and HIV [32].

The role of CAR as a primary receptor is also crucial for Ad-based oncolytic therapy. It is important to acknowledge that CAR expression is often diminished during cancer development. Therefore, many cancer types are resistant to CAR-dependent Ad infection due to profoundly low CAR expression [33,34]. Changing Ad tropism via genetic modification of the viral capsid proteins enables CAR-independent entry [35,36,37,38,39]. Thus, incorporation of an RGD-4C motif into the HI loop of the Ad fiber knob allows the virus to bind to αvβ-class integrins, while replacement of the fiber knob of Ad type 5 with that of type 3 retargets these chimeric viruses (Ad5/3) to the Ad3 receptor desmoglein-2, which is highly expressed in cancer cells [35,40,41,42,43,44]. RGD and Ad5/3 modifications of the fiber remarkably increase the oncolytic efficacy of Ad-vectors in many tumor types, including brain, ovarian, pancreatic, colon cancers, not only in vitro and in vivo but also in clinical application [10,35,36,38,39,40,41,43,45,46,47]. The above approaches for fiber modifications for Ad vector retargeting to a receptor other than CAR can be beneficial for Ad6-based oncolytics as well. It should be noted that Ad vectors with CAR-dependent entry can still be successfully used for several cancer types with high expression of CAR, as it was demonstrated for liver, lung, breast and head and neck cancers [48,49,50,51].

## 4. Trapping of Ad5 and Ad6 by Scavenger Cells and Hepatocyte Transduction

Ad trafficking and receptors used for viral entry into the cells upon systemic delivery drastically differ in comparison to natural infection, and in general, to what we observe in vitro studies, and/or upon local (e.g., intratumoral) Ad injections. Immediately after Ad virions enter the bloodstream, they become coated by many proteins, namely natural IgM, complement proteins, coagulation factors and in case of pre-existing immunity, also adaptive IgG [52]. All these factors can bridge Ads to various cellular receptors, thus having a great impact on final trap location for opsonized Ad particles. The adenoviral hexon as a main target of blood proteins is the most important capsid protein in trafficking upon systemic delivery, although fiber and penton base also play an important role [53,54,55].

The reticuloendothelial system (RES) is the first line of defense against bloodborne coated Ad particles as well as any other immune complexes [56,57,58]. RES is comprised of tissue macrophages and scavenger endothelial cells, which can endocytose foreign particles from blood using their various scavenger, immunoglobulin and complement receptors. At some point RES was renamed to monocyte phagocytic system (MPS), which somewhat neglects the role of pinocytic endothelial cells as scavengers [59]. As both phagocytic and pinocytic cells have been shown to uptake bloodborne viruses, the term RES instead of MPS better describes Ad trapping cells, implying the certain role of scavenger endothelial cells for Ad clearance.

**Figure 3:**
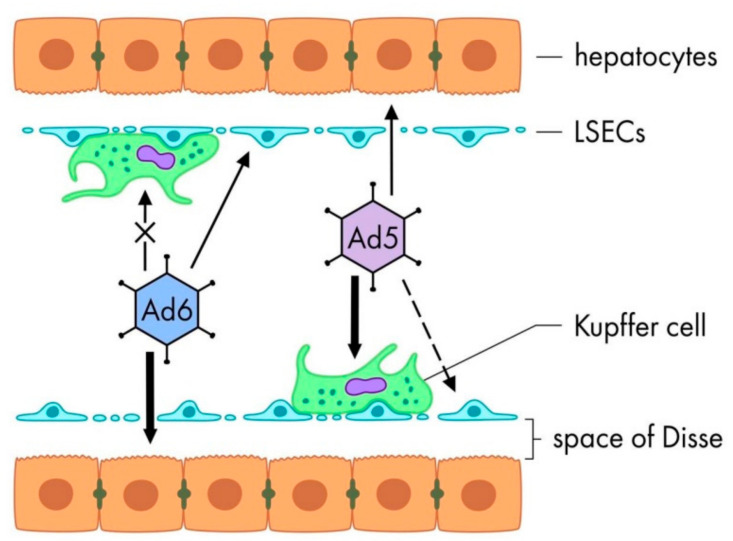
Difference in Ad6 and Ad5 uptake by liver scavenger cells and hepatocyte transduction after systemic delivery. Ad6 can escape Kupffer cells but can be trapped by liver sinusoidal endothelial cells (LSEC), what results in high subsequent hepatocytes transduction. Ad5 is mainly trapped by Kupffer cells and probably by LSEC, which leads to less hepatocyte transduction.

Mammalian species may differ from each other in terms of particular tissue scavengers, which play a dominant role in bloodborne Ad uptake. These differences in RES clearance may explain species-specific toxicity [15]. Thus, it has been shown that in mice, hamsters and non-human primates the main trap for i.v. injected Ad5 is liver scavengers. Among them, Kupffer cells (KC) have been reported to be the primary trap cells in the liver [60,61], but there are also studies showing the dominant role of liver sinusoidal endothelial cells (LSEC) in Ad5 uptake [62] (Figure 3). Adenoviral uptake by KC results in synchronous and rapid KC death, which triggers the innate and adaptive immune responses to the virus and plays a role in inflammation [61]. If the virions are capable to escape liver scavengers, they transduce hepatocytes, which serves as a second dominant mechanism mediating sequestration of bloodborne Ad and also leads to hepatotoxicity in rodents and non-human primates [53,63,64]. Additionally, certain subsets of CD169- and MARCO-macrophages in the marginal zone of the spleen have been shown to be another trap for Ad5 in murine models [65]. Interestingly, in contrast to rodent models, studies in pigs have shown that the lungs, but not the liver, is the organ of main Ad5 uptake suggesting that the main trap of Ad5 in pigs are pulmonary intravascular macrophages (PIM) [66,67].

Ad6 biodistribution profile and trapping has been investigated only in mice and hamsters (to our best knowledge no research on this topic has been performed in other models). It has been shown that Ad6 can escape the uptake by KC in both murine and hamster models, resulting in significantly higher hepatocyte transduction compared to Ad5 (Figure 3) [68,69,70].

Alignment of Ad6 and Ad5 hexon protein sequences revealed that Ad6 has a lower charge (Ad6 displays only 25 negatively and 12 positively charged residues, while Ad5 contains 31 negatively and 17 positively charged amino acids) [70]. These differences in amino acids and overall charge of the hexon protein are located primarily in HVRs, some of which (specifically, HVR 1, 2, 5 and 7) have been shown to be involved in recognition of Ad5 by KC scavenger receptors SR-A [71,72,73,74]. Thus, one of the possible mechanisms of Ad6 escape relies on poor recognition of Ad6 hexon by scavenger receptors on KC. However, given that after systemic injection Ad6 immediately becomes coated by various blood factors, and that KC can use other than scavenger receptors, the escaping mechanisms need further investigation.

The role of the second liver scavenger cells, LSEC, in Ad6 uptake has been shown by Zhang et al. who have shown uptake of Ad6 by LSEC, but not KC [75]. Indeed, LSEC are professional antigen-presenting cells with the high levels of scavenger receptors and high endocytic capacity [58,76]. Importantly, activation or damage of LSEC leads to release of von Willebrand factor (vWF), which binds platelets and collagen with high affinity. This binding can induce thrombocytopenia which is broadly observed after Ad5 administration [15,77]. Therefore, further investigation of LSEC role in Ad6 trapping is of great importance.

In rodent models, Ad6 as well as Ad5 virions that are able to escape scavenger cells (macrophages and/or LSEC), will go to the Disse space and transduce hepatocytes (Figure 3) [24,25]. It has been shown that Ad transduction of hepatocyte is happening via binding of the vitamin K-dependent blood coagulation factors FX, FVII, FIX and FII via their GLA-domain to a central cavity on the Ad hexon, which bridges them to heparan sulfate proteoglycans on the hepatocytes surface [25,78,79]. Different Ad types have been shown to exhibit various binding efficacy to FX, ranging from near picomolar affinity for Ad5 to absence of binding for Ad26 [25]. The current knowledge on the interaction between Ad6 and FX is rather conflicting: while Waddington et al. have shown that binding of FX to Ad5 and Ad6 is approximately the same [25], Khare et al. concluded that FX affinity was tenfold lower with Ad5/6 hexon than that with Ad5 [71]. It’s also unclear how the affinity rate of FX and other blood factors correlate with hepatocyte transduction, as recent data suggested the role of coagulation factors to protect Ad particles against both natural IgM and complement rather than a bridge receptor [80,81].

Ad6 uptake by scavenger cells and subsequent hepatocyte transduction seems to vary among different mice strains, as demonstrated by using the chimeric Ad5/6 vector with HVRs from Ad6 [82]. In this study, Ad5/6 has indeed escaped scavengers and has transduced hepatocytes significantly better than Ad5 in Balb/c mice, while transduction was the same in C57BL/6 murine models. This result is rather unexpected since both strains have the similar levels of KC and LSEC with C57BL/6 macrophages taking up fewer both Ad5 and Ad5/6 virions than BALB/c cells, and Ad5/6 has been shown to evade KC regardless of the mice strain [82]. The further analysis of liver transduction using four mice strains with different Ab numbers has revealed negative correlation of Ad5 hepatocyte transduction with the antibodies level (natural IgM as well as other Ig classes), while in the case of Ad5/6, no negative correlation was detected. This negative correlation observed for Ad5 is logical as the more antibodies mice have, the higher macrophage uptake by SRIg and Ig-receptors will occur leading to the lower final hepatocyte transduction. The absence of that correlation for Ad5/6 has leaded authors to make a suggestion that “most of the biology of Ad5/6 appears to be inverted from that of Ad5”, which could be explained by disruption at some step between Ig binding, complement binding and phagocytic uptake [82].

Taken together, these data indicate that Ad6 trapping by scavenger cells and subsequent hepatocytes transduction essentially differ from that with Ad5. Further studies are needed to clarify the role of LSEC and tissue macrophages in Ad6 and Ad5 uptake as well as exploration of blood factors and receptors on scavenger cells involved in the process of their trapping and liver transduction.

## 5. Seroprevalence of Ad6 and Ad5

Shielding by coagulation factors can protect Ad particles against natural IgM, but does not protect them against adaptive immunoglobulins [83]. It has been shown that anti-hexon IgG is highly effective at blocking Ad5 infection: there are some reports demonstrating that Ad5 can be neutralized by as few as 1.4 anti-hexon IgG antibodies per capsid [84,85]. Consequently, the existing nAb against certain Ad types are often viewed as an obstacle for systemic administration. The high seroprevalence of Ad5 resulting in nearly ubiquitous pre-existing immunity has become the main reason to consider using alternative types.

Fortunately, nAb against different Ad types usually don’t have cross-reactivity; thus, the existence of nAb against one type can be overcome by using the vectors based on another type(s). Ad6 has started to be used as a vaccine and oncolytic platform due to its lower seroprevalence compared to Ad5. Seroprevalence of Ad6 versus Ad5 differs as 66.7–78.8% versus 90.0–94.0% in African countries and Thailand and 8.5–45.7% versus 38.0–69.1% in USA and Europe countries according to different studies summarized by Mennechet et al. [16]. Given that B-cell epitopes are located primarily in HVRs of the hexon protein, which are highly variable between Ad5 and Ad6 [86], it is not surprising that no cross-reactivity between anti-Ad5 and anti-Ad6 nAb has been shown [87]. Moreover, it has been shown that efficacy of Ad6-based vaccine was not affected not only by anti-Ad5 nAb but also by cellular mediated immunity created by previously performed Ad5 immunization of mice [87].

It should be admitted that although Ad6 is less seroprevalent than Ad5, its seroprevalence is slightly higher than that of other types, including commonly used Ad11 and Ad35 (3.0–40.0% and 0–41.0% worldwide, respectively) [16]. Thus, the final choice of a particular type as a platform for gene/cancer therapy development should be made based on the whole body of data on efficacy, safety and seroprevalence.

## 6. Potential and Perspectives of Ad6-Based Vectors as Oncolytics

According to the Wiley database, Ad-based vectors account for 20.3% of all the gene therapy agents against cancer diseases with the Ad5 being the most popular type for oncolytic vector development [1].

Many alternative Ad types, which have less seroprevalence compared to Ad5, have been studied for their oncolytic potential. Most of the original studies on Ad6-based vectors and their use as the potential oncolytic agents were provided by M. Barry’s group. Thus, in their early studies they have tested a panel of four wild-type Ad types: Ad5, Ad6, Ad11 and Ad35 [69]. Ad6 demonstrated oncolytic potential similar to that with Ad5 in prostate, breast, hepatocellular and ovarian cancer cells in vitro [69]. Importantly, Ad6 significantly prolonged the survival time, suggesting Ad6 as a potential type for anticancer treatment [69]. The oncolytic efficacy of wild-type Ad6 was further tested in a broader set consisting of 13 alternative Ad types from species B, C, D and E [88]. Based on the in vitro studies on various human cancer cell lines and the in vivo studies in both immunodeficient mouse and immunocompetent hamster models, Chen et al. have reported the high potential of Ad6-based oncolytic therapies for the treatment of breast, ovarian, kidney and liver tumors [88]. Interestingly, Ad6 was the only type among Ad5, Ad6 and Ad11 that showed significant oncolytic effect in the immunocompetent hamsters bearing subcutaneous kidney cancer HaK cells [88].

The oncolytic potential of Ad6 has been also analyzed in brain cancer models. The wild-type Ad6 vector has been shown to exhibit a strong dose-dependent cytotoxicity against glioblastoma in vitro models. The administration of Ad6 into U87MG subcutaneous xenografts resulted in a significant therapeutic effect, which was comparable to that with Ad5 wild-type control [89]. In addition to tumor size, the ability of Ad6 to destroy the stem cells in glioblastoma tumors has been assessed. It was shown that in treated groups the proportion of stem cells remained the same as in the control group, which indicates that stem cells did not escape the action of both Ad6 and, rather, there was a trend towards a decrease in their proportion in the entire mass of the tumor [89].

The oncolytic efficacy of Ad6 has been also evaluated as a systemic therapeutic in prostate cancer models [90,91]. Ad6 repressed tumor growth after a single intravenous injection, producing a 30-fold less ALT than Ad5 suggesting significantly lower liver toxicity in mice model [90]. Furthermore, it has been shown that chemical shielding with polyethylene glycol may further improve Ad6 safety, albeit it may reduce oncolytic efficacy after intravenous treatment [90]. In another study, Ad6 with a hexon taken from Ad57 (Ad657) was tested as an agent against prostate cancer [91]. Ad657 exhibited–similarly to wild-type Ad6 and Ad5—cytotoxic activities towards prostatic cancer cells in vitro and in vivo after single i.v. administration. Ad57 shares 95% similarity with Ad6 in most genome regions except the hexon protein, and such a difference leads to low cross-reactivity [92,93]. This suggests that Ad657 can be used as a type-switched variant of Ad6 to evade emerging immunity for therapy prolongation. As Ad6 exhibits higher liver transduction than Ad5, Zhang et al. suggested attenuation of Ad6 to avoid hepatocytes and enhanced targeting towards prostatic cells. Four binding sites for liver-specific miRNA-122 were inserted into the 3′-UTR of E1A mRNA to hinder its replication in normal hepatocytes [75]. This recombinant virus, called Ad6miR, has elicited high cytotoxicity in prostate cancer cells in vitro and in vivo while has demonstrated reduced liver transduction in mice model [75].

In contrast to solid tumors, the oncolytic efficacy of wild-type Ad6 against blood cancers has been shown to be low. Senac et al. evaluated the ability of wild-type adenovirus panel (Ad5, Ad6, Ad11, Ad26, Ad40, Ad41 and Ad48) to infect patient-derived multiple myeloma cells. Even though both Ad6 and Ad5 demonstrated intrinsic selectivity towards CD138+ multiple myeloma cells while sparing CD138- normal cells on primary marrow samples from multiple myeloma patients, their cytotoxity on CD138+ were weaker than of Ad26 and Ad48 [94]. Furthermore, the oncolytic activity of Ad6 was considerably lower than that demonstrated with Ad5. Effects on primary follicular lymphoma and leukemia cells were also weak for Ad5 and Ad6 in vitro [95]. Overall, wild-type Ad6 seems not to be an option for blood cancers, which was confirmed in another study with mouse A20 lymphoma cells, which are derived from BALB/c mice and are susceptible to infection and killing by a variety of human Ad types [96]. However, its fiber knob can be replaced with the knob from other types to enhance vector transduction into blood cells as it was performed on Ad5 [37,97]. Moreover, Ad6 without fiber-switching has been shown to be utilized as a cancer vaccine against lymphomas in dogs since it is not necessary for vaccine vectors to infect blood cells [98].

Given the intrinsic ability of Ad6 to avoid KC and transduce hepatocytes, liver cancer seems to be one of the attractive targets for Ad6. However, the oncolytic efficacy of Ad6 against liver cancers has been so far reported only in vitro studies [69,88] and needs further investigation.

The most common approaches to improve the therapeutic potential of Ad-based vectors are often based on the genetic modifications which include: (1) insertion of the therapeutic/imaging transgenes, and (2) targeting cancer cells using tumor-specific promoter or other regulatory elements. This has been widely performed for Ad5-based oncolytic vectors [35,47,99,100,101], but to our best knowledge, it has been rarely applied for Ad6-based vectors (summarized in Table 1). There is a report demonstrating the efficacy of two Ad6 vectors expressing granulocyte-macrophage colony-stimulating factor (GMCSF) and IL-21, but these vectors were used as vaccine adjuvants and not for oncolytic purposes [102]. The lack of recombinant vectors based on Ad6 type might be explained by the fact that in contrast to Ad5, there is no commercially available fast-cloning kits developed for Ad6. This undoubtedly complicates generation of new genetically modified Ad6-based vectors. The utilization of a new approach such as in vitro recombination systems instead of standard in vivo recombination in BJ5183 E. coli strain may promote the development of modified Ad6-based vectors. Thus, Zhang Z. et al. used for vectorization of Ad6 homologous recombination in vitro based on the In-Fusion system [75]. The recent studies from our group have further expanded the use of the In-Fusion system not only for vectorization, but also for inserting transgenes into the Ad6 backbone [103].

## 7. Ad6-Based Vaccines against Cancer and Infectious Diseases

The ability of adenoviral vectors to stimulate the innate immune response, which in turn enhance the immunogenicity of the encoded antigen, make them attractive vectors for vaccine development [104]. The increased attention of scientific community and health providers to Ad-based vaccines against cancer and infectious diseases including measles, hepatitis B and C, rabies, anthrax, Ebola, severe acute respiratory syndrome-1 (SARS-1), human immunodeficiency virus-1 (HIV-1), malaria, tuberculosis, influenza [105] greatly changed the commercial value of the adenovirus-based therapeutics. Not surprisingly, four out of four viral vector vaccines currently approved for full or emergency use against COVID-19 (Vaxzevria by AstraZeneca-Oxford, Sputnik V from Gamaleya Institute, Janssen Vaccine from Johnson and Johnson, and Convidecia by CanSino Biologics) are based on Ad vectors [2,3,4,5].

Ad6 is the promising platform for vaccine development as it has a potential to overcome the pre-existing Ad5 immunity (Table 2 and Table 3). To our best knowledge, Capone et al. have been the first to use Ad6 as a vaccine, creating the MRKAd6-NSmut vector (lately known as Ad6-NSmut) encoding for the hepatitis C virus (HCV) nonstructural region (NS) [87]. The study of MRKAd6-NSmut on rhesus macaques has shown its equivalent potency, breadth and longevity of HCV-specific T-cell responses as the corresponding Ad5-based vector [87].

Importantly, the Ad6-based vector has been shown to overcome both humoral and cellular pre-existing anti-Ad5 immunity and was applied as a booster after Ad5-based vaccine [87]. Clinical trial of Ad6-NSmut singly and in combination with ChAd3-NSmut has revealed the induction of sustained T cell responses of a magnitude and quality associated with protective immunity [107]. The following clinical trial of combination ChAd3-NSmut with Ad6-NSmut as a booster had no significant effect on HCV viral load in patients with chronic hepatitis C viral infection [108]. MRKAd6-NSmut has been also tested in combination with MRKAd24-NSmut vector (based on Ad24 type) and DNA plasmid encoding for NS region as a vaccine for protecting chimpanzees from acute hepatitis induced by challenge with a heterologous virus [106]. Chimpanzees have developed a cross-reactive T-cell response against the challenge virus capable of resolving the infection [106].

The Ad6-based vaccine MRKAd6 has been also evaluated against HIV-1: its clinical trial as a monotherapy and in combination with MRKAd5 has demonstrated the activation of T cell immunity against HIV-1 proteins [109].

Another Ad6-based vaccine against HIV-1 has been constructed based on the so-called single-cycle (SC) vaccines approach, where the deletion of the gene coding for IIIa Ad protein results in production of empty, non-infectious virus particles [115,116]. The preclinical studies in rhesus macaques have demonstrated that while SC-Ad6 vaccine does not protect animals from simian-human immunodeficiency virus upon intramuscular and intranasal routes, the mucosal route resulted in lower viral loads [32].

The further evaluation of SC-Ad6-based vaccine against Ebola in mice, hamsters and rhesus macaques have revealed the induction a high level of serum antibodies in all species and significant protection against pseudo-challenge with a replication-competent vesicular stomatitis virus vaccine expressing the Ebola virus glycoprotein (rVSV-EBOV) in mice and hamsters [31]. The SC-Ad6 has also demonstrated high potential as an anti-influenza vaccine in vivo: SC-Ad vector has generated markedly more influenza virus hemagglutinin protein and require substantially less vector to generate the same immune responses as replicative defective Ad vectors [30].

Adenovirus type 6 has also been utilized as a platform for anti-cancer vaccines (Table 3). Impellizeri et al. have constructed replication deficient Ad6 as a component of an anticancer vaccine expressing human telomerase reverse transcriptase (TERT) [98]. Lately this vector has been studied in humans as a second component of the anti-cancer vaccine V934/V935 with the first component being a telomerase gene-containing plasmid (V934) [117]. A phase I clinical trial of V934/V935 vaccine has showed the successful enhancement of anti-telomerase cellular immunity in human patients with solid tumors [117]. At the same time, a similar vaccine, V930/V932, which contains human epidermal growth factor receptor 2 (HER2) transgene instead of TERT has demonstrated no significant effect on cell-mediated immunity in a clinical trial [118].

Taken together, these data suggest that Ad6 is a promising backbone for the development of vaccines against both cancer and infectious diseases.

**Table 3:** Ad6-based anti-cancer vaccines.

Name/Target	Key Features	Development Status/Combination Regimens	Major Outcomes	Ref
Ad6-dTERT and dTERT-coding DNA plasmid	∆E1, ∆E3; dTERT in E1	Preclinical/dogs with B-cell lymphosarcoma/in combination with COP	No adverse effects; significantly increased overall survival with combination therapy compared to COP alone	[119,120]
Tel-eVax (Ad6-dTERT and dTERT-coding DNA plasmid)	∆E1, ∆E3; dTERT in E1	Preclinical/dogs with malignant lymphoma/in combination with CHOP	No significant adverse effects; generation antibodies against hTERT; significantly increased survival over historic controls	[98]
Ad6-hTERT and hTERT-coding DNA plasmid	∆E1, ∆E3; hTERT in E1	Preclinical/Rhesus monkey/in combination with IMO	No adverse effects; long lasting adaptive immune response	[121]
V935 (Ad6-hTERT) and V934 (hTERT-coding DNA plasmid)	∆E1, ∆E3; hTERT in E1	Phase I clinical trial/Solid tumors patients (38% prostate cancer patients)	Good safety profile; significant increase in cellular responses against hTERT peptide in 1 of 3 pools of peptides	[117]
Ad6- HER2/*neu* and dTERT-coding DNA plasmid	∆E1, ∆E3; human HER-2/neu in E1	Preclinical/Healthy dogs	Detectable and long-lasting adaptive immune response in the absence of autoimmunity or other side-effects	[122]
V932 (Ad6-HER2-CEA) and V930 (two DNA plasmids coding for HER2 and CEA)	∆E1, ∆E3; HER2 and CEA fused to LTB in E1	Phase I clinical trials/Solid tumor patients	No serious adverse events; no measurable cell-mediated immune response to CEA or HER2 in patients	[118]

CEA—carcinoembryonic antigen; CHOP—cyclophosphamide, vincristine, doxorubicin and prednisone; COP—cyclophosphamide, vincristine, prednisone; dTERT—dog telomerase reverse transcriptase; HER2—human epidermal growth factor receptor 2; hTERT—human telomerase reverse transcriptase; IMO—immuno-modulatory oligonucleotide; LTB—B subunit of *E. coli* heat labile toxin.

## 8. Summary

Adenoviral vectors have already proven themselves as effective and safe therapeutics in the fields of oncolytic immunotherapy, vaccine development and genetic diseases. However, the high seroprevalence of conventionally used Adenovirus type 5 has led researchers to expand the range of employed types, favoring those which demonstrate reasonable efficacy in combination with low seroprevalence. Undoubtedly, Adenovirus type 6 is a promising alternative backbone in furthering gene therapy as it can overcome pre-existing anti-Ad5 immunity, and overall, form the strong basis for antitumor drugs and vaccines developments. Even though Ad6 belongs to the same species C as Ad5, it has acquired sufficient differences in its properties, which are mainly dictated by the difference between the Ad6 and Ad5 hexon protein. Namely, Ad6 evades the uptake by liver macrophages and has distinct opsonization patterns by immunoglobulins and perhaps by other blood proteins. This may greatly affect the overall biodistribution of Ad6 after systemic administration. The suggested mechanisms of Ad6 trafficking need deeper investigation. However, using Ad5 and Ad6 as an example, it can be demonstrated how the little difference in virus structure may affect the biodistribution and toxicology profile of a particular Ad type. This serves as a reminder that any new alternative Ad type which is going to be used as a vector for gene therapy, especially when administered i.v., needs to be carefully explored prior to use in humans.

## Figures and Tables

**Table 1:** Ad6-based oncolytic vectors.

Name	Modification	Cancer Type (Model)	Ref.
Wild-type Ad6	None	Human breast, ovarian cancer (in vitro and in vivo nude mice); Human hepatocellular carcinoma (in vitro); Hamster ovary (in vitro); Hamster kidney (in vitro and in vivo Syrian hamsters)	[88]
		Human breast, hepatocellular, ovarian cancer (in vitro); Human prostate cancer (in vitro and in vivo nude mice)	[69]
Ad6-Luc	Wild-type Ad6, CMV-controlled Luc	Human prostate cancer (in vitro and in vivo nude mice), Hamster kidney cancer (in vivo Syrian hamsters)	[90]
Ad657	Ad57-switched hexon	Human prostate cancer (in vitro and in vivo nude mice)	[91]
Ad6miR	miRNA-122-dependent E1A	Human prostate cancer (in vitro and in vivo nude mice)	[75]

CMV—cytomegalovirus promoter; Luc—luciferase gene.

**Table 2:** Ad6-based vaccines against infectious diseases.

Name/Target	Key Features	Development Status/Patients or Model	Major Outcomes	Ref.
MRKAd6-NSmut/HCV	∆E1, ∆E3; HCV NS	Preclinical/Mice, Rhesus Macaques	MRKAd6-NSmut demonstrated equivalent HCV-specific T-cell response as MRKAd5-NSmut with overcoming pre-existing anti-Ad5 immunity	[87]
MRKAd6-NSmut and MRKAd24-NSmut and DNA plasmid/HCV	∆E1, ∆E3; HCV NS	Preclinical/Chimpanzees	Vaccination protected chimpanzees from acute hepatitis induced by challenge with heterologous virus	[106]
Ad6-NSmut and ChAd3-NSmut/HCV	∆E1, ∆E3; HCV NS	Phase I Human Clinical Trial/healthy adults	Good safety profile; priming of CD8+ and CD4+T cell responses by both vaccines, which were sustained up to 1 year	[107]
		Phase I Human Clinical Trial/patients with chronic HCV infection	Good tolerance; no significant effect on HCV viral load	[108]
MRKAd6 HIV-1 trivalent vaccines	∆E1, ∆E3; nef and gag/pol in E1	Phase I Human Clinical Trial/healthy adults	Good safety profile; dose-dependent T cell responses predominantly to Nef, which were durable for at least 52 weeks	[109]
HD-Ad6-Env/HIV-1	HD-Ad5 Ψ and ITRs cross-packaged by Ad6; gp140	Preclinical/Mice, Rhesus Macaques	Overcoming anti-Ad5 immunity; generation of T-cellular and antibodies response but with low neutralization ability; both i.m. and i.vag. routes showed protectivity at different times	[110,111,112]
SC-Ad6-G4 Env and SC-Ad6-F8 Env and SC-Ad657-G4 Env/HIV-1	∆IIIa, ∆E3; G4/F8 gp160 in E3	Preclinical/Mice, Syrian hamsters, Rhesus Macaques	Low overall protection, but notable reductions in SHIV RNA in gastrointestinal tissues in animals that were vaccinated with SC-Ad by i.n. route	[32,113]
SC-Ad6-1157 and SC-Ad6-adjuvant/HIV-1	∆IIIa, ∆E3; HIV-1 gp140/GMCSF/4-1BBL/IL-21/*C. diff* toxin fragment in E3	Preclinical/Mice	Genetic adjuvants improved systemic and mucosal antibody production	[102]
SC-Ad6-TcdA/B/*Clostridium difficile*	∆IIIa, ∆E3; *C. difficile* toxins RBDs in E3	Preclinical/Mice, Syrian hamsters	Strong antibody response; significantly high survival rate after toxin or pathogen challenge over controls	[114]
SC-Ad6-EBOV GP/Ebola virus	∆IIIa, ∆E3; EBOV GP in E3	Preclinical/Mice, Syrian hamsters, Rhesus Macaques	Good antibody response in all animal models; attenuated EBOV-pseudotyped VSV replication in rodents	[31]
SC-Ad6-PR/Influenza virus	∆IIIa, ∆E3; A/PR/8/34 influenza virus HA cDNA in E3	Preclinical/Syrian hamsters, cotton rats	Induced high titer antibodies in both animal models; protectivity in cotton rats	[30]

ChAd3—chimpanzee adenovirus 3; EBOV GP—glycoprotein of Ebola virus; GMCSF—granulocyte-macrophage colony-stimulating factor; gp140, gp160—HIV envelope glycoproteins; HA—hemagglutinin; HCV—hepatitis C virus; HD—helper-depended; HIV-1—human immunodeficiency virus-1; ITR—inverted terminal repeat; NS—nonstructural region; RBD—receptor binding domain; SC—single-cycle; SHIV—simian-human immunodeficiency virus; VSV—vesicular stomatitis virus; Ψ—packaging signal.
